# Plasmid to generate *Mycobacteria* mutants

**DOI:** 10.1186/s13568-018-0537-z

**Published:** 2018-02-01

**Authors:** Qi Deng, Jianzhou Meng, Yan Guan, Yishuang Liu, Chunling Xiao

**Affiliations:** Institute of Medicinal Biotechnology, Chinese Academy of Medical Sciences and Peking Union Medical College, Beijing, China

**Keywords:** Conditional mutant, Tetracycline-inducible expression system, d-alanine:d-alanine ligase, *Mycobacterium tuberculosis*, Plasmid construction

## Abstract

The generation of conditional mutants has been an effective approach to studying bacteria and validating drug targets, and mutants of *Mycobacteria* are no exception. However unlike other bacteria, there is still a paucity of available tools for *Mycobacteria*. We constructed a new plasmid containing tetracycline-repressive expression system (TetRr1.7) and Xer Site-Specific recombinase system to generate label-free controllable expression strains. The plasmid was subsequently used to construct a strain of *M. tuberculosis* expressing the only copy of d-alanine:d-alanine ligase under the control of the tetracycline-repressive promoter. The results showed that the mutant strain lost the ability of colony formation, became more sensitive to d-cycloserine and the cell wall of the mutant strain was disrupted when anhydrotetracycline was added to the medium. Taken together these observations, confirmed that the expression of d-alanine:d-alanine ligase was tightly controlled by the promoter. In conclusion, the new plasmid is a convenient tool for constructing stable conditional mutant strains in *Mycobacteria* and can be used for future target identification.

**Electronic supplementary material:**

The online version of this article (10.1186/s13568-018-0537-z) contains supplementary material, which is available to authorized users.

## Introduction

Tuberculosis (TB) caused by *Mycobacterium tuberculosis* (*Mtb*) still remains a heavy threat to human beings. There were about 10 million patients who developed into tuberculosis and 1 million death every year, many of which were MDR-TB (Kwon 2017). There is an urgent need for new drugs and vaccines to treat the disease. However, the efforts to exploit new anti-TB drugs seemed hopeless as during the last 50 years only two agents were approved (Lu et al. 2014; Blair and Scott 2015). Thus far only a limited number of targets could be used to develop the drugs, which explains the low success rate. Further the identification of new targets was difficult because of the lack of tools to research the molecular microbiology of *Mtb*.

The plasmids used for molecule manipulation in *Mycobacteria* limited to pMind, pMV261, pMY769, pAZI9479, pNIL-pGOAL and so on. Plasmids pMind, pMV261 and pMY769 can carry the DNA fragment into cell, but the gene dosage cannot be quantified as these plasmids cannot integrate into the genome or another copy of the gene exists in the host genome and cannot be controlled by the operon of the plasmid. Plasmid pAZI9479, a suicide plasmid for *mycobacteria*, can replace the promoter of the target gene with the ptr promoter through homologous recombination. However the mutant strain is not stable and can reverse to the wild type. Plasmid pNIL-pGOAL can knockout a target gene through double homologous recombination. However, in our hands, knockout strains of the essential genes could not be obtained and the target genes were cutoff so we could only study at the deficiency conditions. Inducible systems used in *mycobacteria* are also problematic. Currently, the mainly reported systems contain: acetamidase inducible system, pristinamycin inducible system, IPTG inducible system, nitrilases inducible system, theophylline riboswitch system and tetracycline inducible system. The acetamide-inducible system is the earliest mycobacterial inducible system described, which is used for over-expression, but the basal activity of promoter is high and prone to recombination (Parish et al. 1997; Brown and Parish 2006). Pristinamycin inducible system, IPTG inducible system are also successfully developed for gene regulation in *mycobacteria* (Forti et al. 2009; Ravishankar et al. 2015). IPTG inducible system is more frequently used in *E. coli* expression and follow-up applications in *mycobacterium* are limited. There are also references about nitrilases inducible system and theophylline riboswitch system (Pandey 2009; Seeliger et al. 2012). Tetracycline inducible system is the most widely used and has been validated that they can be used to regulate gene expression in animal models of infection (Carroll et al. 2005; Ehrt et al. 2005a, b; Hernandez-Abanto et al. 2006). Considering all the above, we endeavoured to design a new plasmid containing tetracycline inducible system with the following advantageous characteristics that allow the researcher to: (i) construct a stable mutant strain; (ii) control gene expression quantitatively through the promoter of the plasmid.

Herein we describe the successful construction of a new plasmid containing a TetRr1.7, a tetracycline-repressive expression system which has been shown to express a target gene expression quantitatively in the presence of the repressor (tetracycline or anhydrotetracycline) (Guo et al. 2007). Target gene expression was under the control of a promoter which could bind with the TetRr1.7- anhydrotetracycline complex. Herein we also validate the plasmid and illustrate that we could indeed regulate the gene expression of d-alanine:d-alanine ligase (Ddl) in *Mycobacteria*.

## Materials and methods

### Bacterial strains and media

*Mycobacterium tuberculosis* H_37_Rv (ATCC27294) was cultured on Middlebrook 7H10 agar media supplemented with Oleic Albumin Dextrose Catalase (OADC) (Allen 1998; Hodgkinson et al. 2015) or in 7H9 broth plus OADC and polysorbate 80. *Escherichia coli* DH5α (TransGen Biotech, Beijing, China) was cultured in Luria–Bertani (LB) broth or on LB agar medium. Kanamycin (Amresco, Bedfordshire, UK) was added at the concentration of 100 µg mL^−1^ for *E. coli* and hygromycin (Amresco, Bedfordshire, UK) 100 µg mL^−1^ for *Mycobacteria*. Anhydrotetracycline (Sigma-Aldrich, Germany) was added as appropriate. Plasmids used are listed in Table 1.

**Table 1:** Plasmids used in this study

Plasmids	Application	Source
pMind	Used to construct pMDX and as template to amplify hygromycin resistance gene	Robertson, Brian D (Blokpoel et al. 2005)
pJRD215	Used as template to amplify streptomycin resistance gene	Professor Xiangmei Liu (Chen et al. 2011)
pMY769	Used as template to amplify gap fragments	Professor Francesca Forti (Forti et al. 2009)
pGOAL19	Used as template to amplify the promotor pAG85	Tanya Parish (Parish and Stoker 2000)
pEGFP-C1	Used as template to amplify EGFP gene	Doc Zhang (Zhang et al. 1996)
pJV53	Used to facilitate recombination	van Kessel, J.C. (van Kessel and Hatfull 2007)
pMDX	The plasmid tool	This study

### Plasmid constructions

Genomic DNA was extracted from *Mtb* H_37_Rv in the logarithmic phase and used as PCR template, as previously described (van Helden et al. 2001). PCR conditions were as follows: hot start at 94 °C for 10 min, followed by 30 cycles of 94 °C for 40 s, 60 °C for 30 s, and 72 °C for 30–120 s (depending on the fragment length), and a final extension at 72 °C for 10 min. Plasmid pMind was digested with *Nhe*-I to remove pAL5000 fragment, the replicon of *Mycobacteria*, and to obtain pIMBS. Promoter *P*_*myc1*_*tetO* (Ehrt et al. 2005a, b) was chosen to control the target gene expression in *Mycobacteria* and was synthesized chemically by Ruibiotech (Beijing, China). *P*_*myc1*_*tetO* and pIMBS were digested with *Hin*d-III and *Spe*-I. After ligation pIMBSt was obtained and which also no longer contained the hygromycin resistant gene. The streptomycin resistance gene was amplified using primers SmdifS and SmdifR from pJRD215, and cloned between the *Xba*-I and *Bam*H-I sites of pIMBSt to get pIMBSs in which two dif sites of *Mtb* lineated in the primers that were added to excise the resistance gene in *Mycobacteria*. Then the hygromycin resistance gene was amplified from pMind using HygroS and HygroR primers to substitute the streptomycin resistance, obtaining pIMBSh in which the hygromycin resistance gene was controlled by the promoter *Ptet2*. A fragment about 370 bp of pMY769 was amplified using primers Gap1S and Gap1R, and the product was cloned into the *Bam*H-I and *Spe*-I sites of pIMBSh to separate the P_myc1_tetO and the downstream operon, obtaining pIMBSg. Primers P85S and P85R were used to amplify promoter *pAG85* from pGOAL19, while PeS and PeR amplified eGFP from pEGFP-C1. Then, both PCR products were mixed as templates and primers P85S and PeR were used to synthesize a whole fragment containing promoter and eGFP. The fragment was cloned using *Pfl*m-I and *Spe*-I sites of pIMBSg to obtain pIMBSe. Primers gap2S and gap2R were used to amplify fragment from pMY769 and the product was cloned into *Spe*-I digested pIMBSe through seamless cloning, getting pIMBSeg. Part of the TetRr 1.7 was synthesized and cloned using the *Xba*-I and *Kpn*-I sites of pIMBSeg to generate pIMBSr. Primers PfurA102S and PfurA102R were used to amplify promoter *PfurA102* from pMY769, and cloned into pIMBSr digested by *Xba*-I through seamless cloning, thereby obtaining pMDX. All primer sequences used above are listed in Additional file 1.

### Construction of the promoter replacement mutants in Mtb

To construct *Mtb Ddl* mutant strain, the forward fragment and fragment after initiation codon ATG (about 400 bp) of the target genes were separately amplified by primers d1S, d1R and d2S, d2R (listed in Additional file 1) and then cloned into pMDX to construct pMDXD. The competent *Mtb* cells and plasmids were prepared as described (Hinds et al. 1999; Parish et al. 1999). In brief, the cells were collected and washed three times with 10% glycerol reducing the volume each time. They were then suspended in 1/500 of the initial volume using ice-cold 10% glycerol. In order to transform the competent cells, 5 µL pJV53 (no more than 1 µg) was added to 200 µL competent cells, and transferred to a 0.2 cm cuvette (Bio-rad, Hercules, CA, USA). The electroporation program used was: voltage 2.5 kV, capacitance 25 µF, resistance 1000 Ω, and a pulse time of 15–25 ms. The pulsed cells were cultured in 5 mL 7H9 broth for 24–48 h at 37 °C, spread on 7H10 agar containing 100 µg mL^−1^ kanamycin and incubated at 37 °C until bacterial colonies formed. The positive strain was confirmed by colony PCR using primer kmS (see Additional file 1) and primer kmR (see Additional file 1). The Mtb:pJV53 were cultured in 7H9 broth, containing 20 µg mL^−1^ kanamycin and 0.2% (w/v) succinate at 37 °C, and 0.2% (w/v) acetamide was added to the culture for another 24 h (van Kessel and Hatfull 2007). The competent cells were prepared as before and electro-transformed with *Hin*d-III and *Pci*-I digested pMDXD. The pulsed cells were refreshed and then spread on the 7H10 agar containing 100 µg mL^−1^ hygromycin and incubated at 37 °C until bacterial colonies formed. The bacteria were subsequently transferred to 7H9 liquid medium without antibiotic and incubated at 37 °C to allow them to excise the hygromycin resistance gene. The genome of the mutant was sequenced to ensure the successful insertion of the DNA fragments into the *Mtb* genome. The positive mutant strain was named Mtb::*Ddl*.

### Mutant colony formation

The mutant strain and wild-type strain were cultured separately in 7H9 broth at different concentrations (0, 2, 20, 200, 2000 ng mL^−1^) of anhydrotetracycline. Until OD_600_ reached 0.55, the bacterial suspensions were diluted 100 times and then spotted on 7H10 agar media in six-well plates. The plates were incubated at 37 °C to observe colony formation.

### Alterations in cell-wall contents of the mutant

The mutant strain was cultured in 7H9 medium in the presence of different concentrations of anhydrotetracycline (0, 2, 20, 200, 2000 ng mL^−1^) for 2 weeks. The wild type strain was cultured in 7H9 medium with d-cycloserine added at the concentration of 12 μg mL^−1^ for 2 weeks. Cell wall of *Mycobacteria* was extracted as described previously (Besra 1998; Meng et al. 2015).

### MIC of d-cycloserine to the mutant

The minimum inhibitory concentration (MIC) was determined using the broth dilution method. Briefly, the mutant strain and wild-type strain were cultured in 7H9 medium in 96-well microtiter plates containing different concentrations of anhydrotetracycline (0, 2, 20 ng mL^−1^). A two-fold serial dilution of d-cycloserine was added, giving final concentrations of 128, 64, 32, 16, 8, 4, 2, 1, 0.5 and 0.25 μg mL^−1^. Rifampicin and isoniazid were used as control and the final concentrations of these two drugs were 64, 32, 16, 8, 4, 2, 1, 0.5, 0.25, 0.125, 0.0625 and 0.03125 μg mL^−1^.

### The genetic stability of the mutant

The mutant was cultured in 7H9 broth and inoculated once every 10 days. After fifty generations, the genome was extracted and the segment we inserted was sequenced.

## Results

**Fig. 1:**
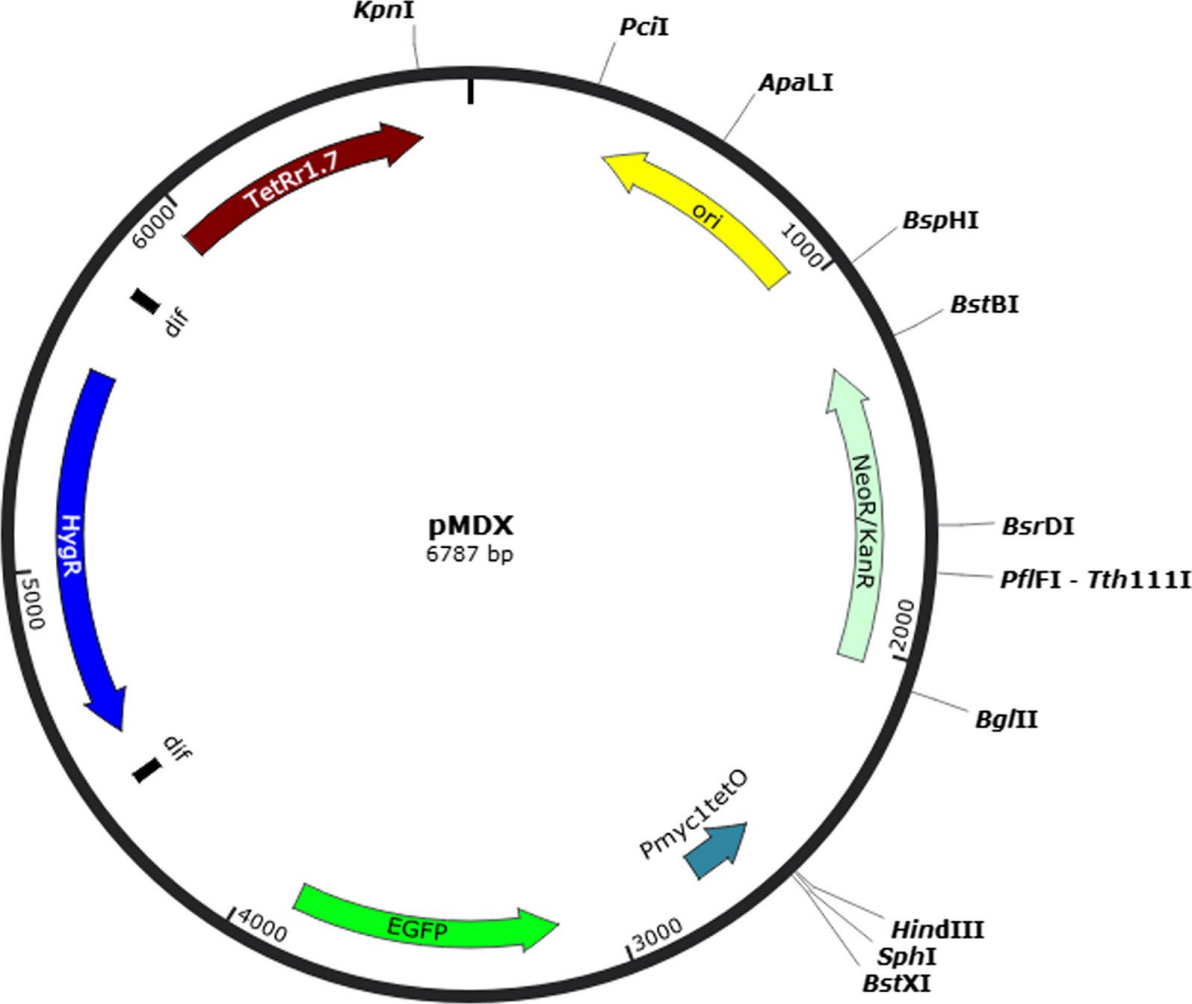
The map of the pMDX

The plasmid pMDX with illustrated operon and restriction sites is shown in Fig. 1. To control the expression of the target gene in *Mtb*, its upstream sequence should be cloned at the *Kpn*-I site anticlockwise, and the downstream sequence anticlockwise at *Bst*-XI site, with the ATG initiation codon of the gene at *Bst*-XI site. The lineated plasmid can conduct homologous recombination twice to substitute the promoter of the target gene with *P*_*myc1*_*tetO*, and the expression of *P*_*myc1*_*tetO* is controlled by the repressor TetR-r1.7. The expression of target gene can therefore be repressed by anhydrotetracycline.

### Colony formation efficiency of the mutant

**Fig. 2:**
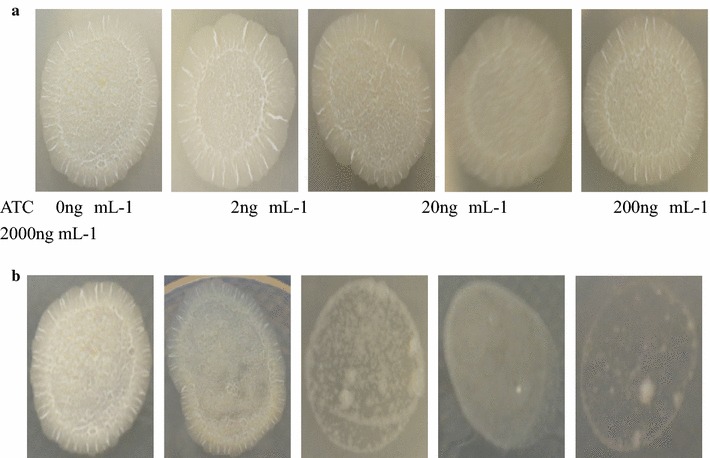
Bacterial colony formation of the wild-type strain (**a**) and mutant strain (**b**) at different concentration of anhydrotetracycline

Given that the *Ddl* gene is essential for *Mycobacterium*, the ability to form bacterial colonies was investigated in order to verify that the expression of *Ddl* was controllable. Different concentrations of anhydrotetracycline were therefore added to the culture of Mtb::*Ddl* strain harbouring the pMDXD plasmid, and colony formation on 7H10 agar media was recorded. Mutant and wild-type strains were equally efficient at forming colonies in the presence of 0–2 ng mL^−1^ anhydrotetracycline (Fig. 2). At 20 ng mL^−1^ anhydrotetracycline, the mutant strain could form colonies but there were fewer colonies than for the wild type strain (Fig. 2). And, strains cultivated in media containing 200 and 2000 ng mL^−1^ anhydrotetracycline formed more less bacterial colonies (Fig. 2). The results show that anhydrotetracycline did not affect the colony formation of wild type *Mycobacterium.* Also the mutant strain can form colony without anhydrotetracycline, but colony formation is impeded with increasing concentrations of anhydrotetracycline. These data indicate that expression of *Ddl* decreases when anhydrotetracycline is added.

### Alterations in the cell wall of the conditional mutant

**Fig. 3:**
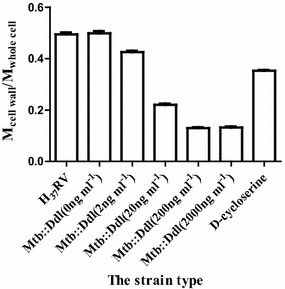
The proportion of the cell wall mass to the whole cell mass. The whole cell wall was extracted from wild-type H37Rv and Mtb::*Ddl* cultured in the presence of 0, 2, 20, 200, 2000 ng mL^−1^ anhydrotetracycline

*Ddl* catalyzes the ATP-driven ligation of two d-alanine (d-Ala) molecules to form the d-alanyl:d-alanine dipeptide, a key building block in peptidoglycan (Bruning et al. 2011). Peptidoglycan is critical to the integrity of the bacterial cell wall. It was therefore inferred that suppressing the expression of *Ddl* will result in the disruption of the bacterial cell wall. d-cycloserine is a second-line anti-TB drug, the mechanism of which is to block peptidoglycan synthesis. In theory, the effect of anhydrotetracycline to the mutant strain is similar to the effect of d-cycloserine to the wild type strain. Thus, we also treated *Mtb* H_37_Rv with a sublethal concentration of d-cycloserine. The whole cell wall was extracted from *Mtb* H_37_Rv and Mtb::*Ddl* in the plateau growth phase cultured in 7H9 containing anhydrotetracycline or d-cycloserine. The ratio of cell wall mass to whole cell mass of both strains is shown in Fig. 3. Without anhydrotetracycline, the mutant cell wall content was similar to that of the wild-type. The ratio of cell wall mass to whole cell mass reduced with increasing concentrations of anhydrotetracycline. For example, a ratio of 0.13 when 200 ng mL^−1^ anhydrotetracycline was added to the culture suggests that the mutant cells lost an average of 73.7% cell wall materials. When 12 μg mL^−1^ d- cycloserine was added, the ratio of cell wall mass to whole cell mass was 0.35, which indicated that an average of 35% cell wall was lost. These data confirmed that the expression of *Ddl* can be regulated using anhydrotetracycline, affecting Ddl protein level. Treated the wild type strain with d-cycloserine could have the same effect with treated the mutant strain with anhydrotetracycline.

### MIC of d-cycloserine to the mutant

The MIC of d-cycloserine to the mutant Mtb::*Ddl* was determined, as d-cycloserine is a known inhibitor of Ddl. The MIC of d-cycloserine to the mutant was twice the MIC to wild-type strain. This may because the promoter *P*_*myc1*_*tetO* was stronger than its original promoter. The MIC of d-cycloserine to the mutant almost remained unchanged when the concentration of anhydrotetracycline was 2 ng mL^−1^. When 20 ng mL^−1^ anhydrotetracycline was added to the media, the MIC of d-cycloserine to the mutant was 1 μg mL^−1^, while the MIC to wild type strain was 16 μg mL^−1^. The MICs of rifampicin and isoniazid to the mutant were both 0.0625 μg mL^−1^ when 0, 2 and 20 ng mL^−1^ anhydrotetracycline was added, which were the same to the MICs of rifampicin and isoniazid to wild type strain. These data confirmed again that the anhydrotetracycline can repress the expression of *Ddl*.

### The genetic stability of the mutant

Genetic stability is one of the shortcomings of the existing plasmids for gene manipulation in *Mycobacteria*. To test whether the mutant can be genetic stable, the mutant was subcultured and the segments we inserted of the fiftieth generation was sequenced. The result confirmed that the mutant gene remained unchanged.

## Discussion

Developing tools for conditional gene regulation is of great importance to gene function research and drug target identification in bacteria. Experimental control of target gene expression is desired and can be achieved by replacing the native promoter of the target gene with a tightly regulated promoter whose activity can be controlled by the researcher (Gomez and Bishai 2000; Chalut et al. 2006; Gandotra et al. 2007). To date, the plasmids for promoter replacement in *Mycobacteria* are limited and have disadvantages. In this study a new plasmid, pMDX, was constructed which can be used to generate a stable mutant strain and control gene expression quantitatively as demonstrated using d-alanine:d-alanine ligase(Ddl) as a subject for target gene regulation in *Mycobacteria*.

The plasmid pMDX contains a regulated mycobacterial expression system TetRr-1.7 that permits the silencing of a gene by the addition of anhydrotetracycline to the culture medium. Regulation of gene repression by TetR-r1.7 has been validated through silencing secA1 in *Mycobacterium smegmatis* (Guo et al. 2007) and prcBA in *M. tuberculosis* (Gandotra et al. 2007). As high-level TetR expression is required when repression occurs, the promoter *PfurA102* was used to control the expression of TetR-r1.7. The target gene was operated by *Pmyc1tetO* containing two tetO2 which can bind to TetRr1.7-anhydrotetracycline complex to shut down gene expression. For mutant strain screening, the hygromycin resistance gene was introduced into the plasmid, but it was nulled after obtained the mutant strains. This screening marker would otherwise lead to erroneous judgements especially during inhibitor screening and can affect the growth of the bacteria. Hence two dif fragments were added to both sides of the resistance gene, which were recognized and cut-out by Xer recombinases, obtaining the label-free mutant strains (Cascioferro et al. 2010). The eGFP gene was also cloned into the plasmid to monitor the growth of the bacteria in macrophage by detecting its fluorescence. This function still needs to be validated in a further study.

Another obstacle for conditional mutant strain construction in *Mycobacteria* is the low rate of homologous recombination. Gordhan and Parish described three methods to pretreat DNA to enhance the homologous recombination (Gordhan and Parish 2001). Single-stranded phagemid DNA can abolish unwanted recombination but the problem is the generation of sufficient amount of single-stranded DNA especially for the GC rich *Mtb* genome. UV pretreated DNA can raise the homologous recombination events, however, additional point mutations are inevitable. Alkali-denaturation will lead to no additional mutations but lower the homologous recombination events. Lineated double-stranded DNA may also cause higher homologous recombination probability, but lineated DNA is not stable in bacteria. Plasmid pJV53, an *Escherichia*–*Mycobacteria* shuttle plasmid, can generate Che9c mycobacteria phage gp60 and gp61 proteins, which are homologous to recombinant protein RecE and RecT respectively. These recombinant proteins promote foreign genes to recombine with homologous genes in *Mycobacteria* with high efficiency (van Kessel and Hatfull 2007). In this study, before the lineated DNA was introduced into *Mtb*, plasmid pVJ53 was therefore electroporated into *Mtb* to improve homologous recombination. Only the double cross-over mutants could form colonies on the 7H10 agar media, the single cross-over mutants were not viable.

To verify the effective application of the new plasmid in gene regulation, we chose *Ddl* as the reporter gene. The results indicated that the growth of the mutant strains was comparable to wild type strain without anhydrotetracycline. Further, the growth of the mutant strains was abolished when anhydrotetracycline was added as a result of the reduced expression of *Ddl*. *Ddl* gene repression was positively correlated with the concentration of anhydrotetracycline. When 2 ng mL^−1^ anhydrotetracycline was added, the cell wall mass of the mutant decreased by only 14.7% and there was no difference between the MIC of d-cycloserine to the mutant with or without this effector. However, when the concentration of anhydrotetracycline was increased to 20 ng mL^−1^, the cell wall mass decreased to 44.9% and the MIC was 16 times lower. Cell wall mass was only 26.2% when 200 ng mL^−1^ anhydrotetracycline was added. After adding 2000 ng mL^−1^ anhydrotetracycline hardly any colonies were formed and cell wall mass did not changed from 200 ng mL^−1^. It was speculated that the repression of *Ddl* gene expression through the addition of anhydrotetracycline had reached its limit. Given the low copy number at 200 and 2000 ng mL^−1^ anhydrotetracycline, the MIC of d-cycloserine to the mutant was not determined. The data confirm that we were able to fully repress D*dl gene* expression using anhydrotetracycline, and that the amount of Ddl protein correlates to the concentration of anhydrotetracycline added to the culture medium.

This study successfully constructed a new plasmid pMDX for generating stable conditional mutant strains in *M. tuberculosis* using lineated double-stranded DNA electro transformed cells. The application of this plasmid and prospects were demonstrated as we were able to conditionally regulate gene expression of *Ddl*. However, this novel approach will need further validation as it is not conform classic molecular manipulation.

## Additional file

**Additional file 1.** PCR primers used in this study.

## Abbreviations

ATCC: American type culture collection
Ddl: d-alanine:d-alanine ligase
LB: Luria–Bertani
MIC: minimum inhibitory concentration
*Mtb*: *Mycobacterium tuberculosis*
OADC: Oleic Albumin Dextrose Catalase
TB: tuberculosis
