# Supplementary material for: Plasmid to generate Mycobacteria mutants
Source: AMB Express. 2018 Feb 1;8:13. doi: 10.1186/s13568-018-0537-z (PMC5794715; doi:10.1186/s13568-018-0537-z)
Supplement: Supplementary file 1 — Additional file 1. PCR primers used in this study. [file 13568_2018_537_MOESM1_ESM.docx]

**Applied Microbiology and Biotechnology Express**

**Plasmid to generate Mycobacteria mutants**

Qi Deng^1^, Jianzhou Meng^1^^[[1]](#footnote-1)^,Yan Guan^1^, Yishuang Liu^1^, Chunling Xiao^1^

1. Institute of Medicinal Biotechnology, Chinese Academy of Medical Sciences and Peking Union Medical College, Beijing, China.

**Table S1.PCR primers used in this study：**

| **Primers used** | **Sequence** | **Use** |
| --- | --- | --- |
| SmdifS | TCGATCTAGATAAGCCGATAAGCGACATTATGTCAAGTTAATGCGGTAGTTTATCAC | To amplify streptomycin resistance gene from pJRD215 |
| SmdifR | ACTGGGATCCACTTGACATAATGTCGCTTATCGGCTTACCTGTTTGGGGTCGTTTGC | To amplify streptomycin resistance gene from pJRD215 |
| HygroS | CAGAAAACTGAAGGAACCTCCAGTGACACAAGAATCCCTGTTACTTC | To amplify hygromycin resistance gene from pMind |
| HygroR | GATAGGAGAAGTCGCTTGATATTTAGGCGCCGGGGGCGGTGTCTG | To amplify hygromycin resistance gene from pMind |
| Gap1S | CTAGGATCCTATCCTGATTCAGGTG | To amplify a fragment about 370 bp of pMY769 |
| Gap1R | CCTACTAGTCCATAGGATGGCAAGATC | To amplify a fragment about 370 bp of pMY769 |
| P85S | GCTTCCATCCTATGGCGACACATGCCCAGACACTGCG | To amplify promoter *pAG85* from pGOAL19 |
| P85R | CTCGCCCTTGCTCACCATTCTTGCTTCCCTCATCCTC | To amplify promoter *pAG85* from pGOAL19 |
| PeS | ATGGTGAGCAAGGGCGAGGAGCTG | To amplify eGFP from pEGFP-C1 |
| PeR | GCTACTAGT*T*GCGTACTCGCCCGGCCCGCAA*C*ATCGGCCCGGGTGATTTAACAAAAATTTAACGCGAATTTTAACAAAATATTAACGCTTACAATTTATGAGTCCGGATTACTTGTACAGCTCGTC | To amplify eGFP from pEGFP-C1 |
| gap2S | CTCCCACTAGTCTAATCAGAATTG | To amplify a fragment from pMY769 |
| gap2R | CGAGTACGCAGAGGTCTGCCTCGTG | To amplify a fragment from pMY769 |
| PfurA102S | CTTATCGGCTTACCATCCTGACGGATGG | To amplify promoter PfurA102 from pMY769 |
| PfurA102R | CTTTTATCTAATCTAGACATCATCGCGGCCGC | To amplify promoter PfurA102 from pMY769 |
| d1S | CCTGCCATGGTGGTGGTGCATCATCATCATCATCACAGTGCTAACGAC | To amplify the forward fragment of *Ddl* |
| d1R | CAGTGCATGCGGGTTTGACGAACACCGGT | To amplify the forward fragment of *Ddl* |
| d2S | GGGACATGTTTTGGCGAACGCCTG | To amplify the downstream fragment of *Ddl* |
| d2R | GAAGGTACCAACCTGCAGAGGCTACCGGGTC | To amplify the downstream fragment of *Ddl* |
| kmS | CCGCGATTAAATTCCAACATGGATG | Primer for colony PCR to confirm mutant strain |
| kmR | AATGAAACTGCAATTTATTCATATC | Primer for colony PCR to confirm mutant strain |

1. Corresponding author: Dr.Meng Tel/fax: +86 10 63020226. Email address: mengjianzhou@126.com [↑](#footnote-ref-1)
